# NICE guidance on the use of carmustine wafers in high grade gliomas: a national study on variation in practice

**DOI:** 10.3109/02688697.2012.673651

**Published:** 2012-05-28

**Authors:** Stephen J. Price, Ian R. Whittle, Keyoumars Ashkan, Paul Grundy, Garth Cruickshank, On behalf of the UK-HGG Study Group, members listed at the end

**Affiliations:** 1 Department of Clinical Neurosciences, University of Cambridge, Addenbrooke’s Hospital, Cambridge, UK; 2 Department of Clinical Neurosciences, University of Edinburgh, Western General Hospital, Edinburgh, UK; 3 Department of Neurosurgery, King’s College Hospital, London, UK; 4 Department of Neurosurgery, Wessex Neurological Centre, Southampton University Hospitals Trust, Southampton, UK; 5 Department of Neurosurgery, The University of Birmingham, Queen Elizabeth Hospital Neuroscience Centre, Edgbaston, Birmingham, B15 2TH, UK

**Keywords:** Brain neoplasms, implementing NICE guidance, carmustine wafers, improving outcomes guidance

## Abstract

*Background*. Multidisciplinary team (MDT) working in oncology aims to improve outcomes for patients with cancer. One role is to ensure the implementation of best practice and National Institute for Health and Clinical Excellence (NICE) guidance. In this study, we have assessed the role of MDT in implementing the TA121 appraisal of the use of carmustine wafers in high grade gliomas. *Methods*. 296 patients with high-grade glioma suitable for maximal resection were recruited from 17 Neurosurgical Centres. The number of patients treated with carmustine wafers and reasons for not using this were recorded. Complications at 48 hours post-operatively and at 6 weeks post-radiotherapy were recorded. *Results*. 94/296 (32%) of suitable patients received carmustine wafers. In 55% of cases carmustine was not used due to either surgeon preference or a lack of an MDT decision. There was no increased complication rate with carmustine use at either 48 hours post-surgery or at 6 weeks post radiotherapy. Use of carmustine wafers did not decrease access to and use of chemoradiotherapy. *Conclusions*. One third of patients suitable for carmustine wafers received them. Their use was neither associated with more frequent complications, nor decreased use of chemoradiotherapy. Implementation of NICE TA121 Guidance is extremely variable in different MDTs across the United Kingdom.

## Introduction

Multidisciplinary team (MDT) working is central to the modern management of cancer patients. MDTs were introduced to help overcome the shortfalls of cancer care in the United Kingdom and were important parts of both the Calman – Hine report^1^ and the NHS Cancer Plan produced in 2000.^2^ The role of MDTs was three-fold:

1. To ensure that all aspects of diagnosis, treatment and care are provided by designated specialists, working together effectively in multidisciplinary teams;
2. To ensure that care is given according to recognised guidelines;
3. To ensure that mechanisms are in place to support entry of eligible patients into clinical trials.^3^

The publication of the ‘Improving Outcomes Guidance by the National Institute for Health and Clinical Excellence (NICE)’ for the different cancer sites has been the major driver in ensuring MDT working.^4^ The guidance for CNS tumours was published in 2006, and it outlines the importance of discussing all patients pre-operatively in a MDT setting to ensure the best surgical treatment for these tumours. The MDT is also meant to ensure treatment accords to recognised guidelines – including implementing NICE guidance. There has been little published on the effectiveness of MDTs^4^; there are even fewer studies looking at the implementation of NICE guidance by MDTs.

High grade gliomas are the commonest intrinsic brain tumours and account for more average years of life lost than all the commoner cancers.^5^ It has become the commonest cause of cancer death in men under the age of 45 and women under the age of 35. Although surgical resection can greatly reduce tumour bulk, complete excision is virtually impossible due to the infiltrative nature of these tumours. Although adjuvant radiotherapy and chemotherapy improves survival, death is inevitably from either recurrent or progressive disease.

In an attempt to treat the infiltrating tumour cells, there has been much interest in using local therapies inserted at the time of surgery. Carmustine wafers (Gliadel^®^) are biodegradable polymers that release 7.7 mg of carmustine over a few weeks directly into the resection cavity. A phase III study in newly diagnosed high grade gliomas showed that carmustine wafers were well tolerated and associated with a survival advantage.^6^ NICE issued guidance on the use of carmustine wafers for the treatment of newly diagnosed gliomas in June 2007 (NICE Technology Appraisal Guidance TA121).^7^ The guidance recommended the use of carmustine wafers as an option for the treatment of newly diagnosed high grade gliomas (WHO Grade III and IV) in patients where it is felt that 90% of the tumour could be resected, and who are treated in specialist centres that conform to guidance in ‘Improving outcomes for people with brain and other central nervous system tumours’.^8^ Multidisciplinary teams should be used to enable preoperative identification of patients in whom maximal resection is likely to be achieved.

The primary aim of this study is to assess the variation of implementation of the NICE technology appraisal concerning carmustine wafers by MDTs throughout the United Kingdom and explore the reasons for not using this treatment option. Secondary aims were to evaluate morbidity associated with carmustine wafer insertion and whether patients having this treatment were denied access to other oncological therapies for their brain tumour.

## Methods

All centres who conform to the guidance ‘Improving Outcomes for people with brain and other central nervous system tumours’ across the United Kingdom were approached by letter to participate in the study. REC approval (South West Research Ethics Committee 08/H0206/35) was sought and local Trust R&D approval was obtained in all participating centres.

MDT teams identified eligible patients who met the inclusion criteria during routine MDT meetings. Eligible patients included those aged ≥ 18 years at the time of diagnosis with a radiological diagnosis of probable high grade glioma (WHO grade III or IV) where a maximal resection (90% or more) was judged likely to be possible. Data was recorded prospectively by the nominated study researcher in each centre (Neurosurgeon, Neurooncologist or Neurooncology specialist nurse) for patients who received surgery between February 2008 and October 2010. Data collected was anonymised-coded for each patient, by the assignment of a unique patient identification number to each record. Data was recorded post-operatively on the details of the surgical procedure and then at 48 hours post-surgery. All tissue was categorised according to the WHO system by a local Consultant Neuropathologist. There was no central pathology review. The patient was followed up at 6 weeks post-radiotherapy by the researcher. Anonymised data was transferred to the dedicated study database. Analysis was undertaken in MS Excel and SPSS for Windows.

## Results

### Centres contributing to study

Seventeen of the 31 (55%) neurosurgical centres in Great Britain screened 307 patients. The catchment areas of these units cover a total population of approximately 32 million people. Of the 307 patients screened, 11 were deemed ineligible since 8 underwent surgery prior to the study period, 2 were not newly diagnosed patients, and 1 patient refused surgery. The centres recruiting patients are listed at the end of the paper. Four centres contributed 64% of the sample and 62% of the carmustine patients.

### Recruited cohort

There were 197 males (67%) and 99 females (33%) with a median age of 61 years (interquartile range 53 – 66). All patients were over the age of 18, and all were diagnosed with high grade gliomas: 260 (88%) with glioblastomas, 7 (2%) with gliosarcomas and 27 (9%) with WHO Grade III tumours. A histological diagnosis was not available in 2 cases. 94% of the total number of tumours were in the supratentorial compartment and 169 (57%) in the right hemisphere with 93% having a single focus and 20 (7%) crossing the midline.

The Karnofsky Performance Status (KPS) was recorded in 289 patients and the median of the sample was 90. Ninety-one percent of the sample had a KPS of ≥ 70, 78%≥80 and 56%≥90. The WHO performance status (recorded in 289 patients) was 0 in 111 patients (38%), 1 in 127 (44%), 2 in 38 (13%), 3 in 7 (2%) and 4 in 6 (2%) patients.

A total of 285 patients (96%) had a pre-operative assessment that > 90% of the tumour could be resected. In the remaining cases, five needed urgent surgery, and in six cases, the data was not recorded. This decision was made on the basis of pre-operative MRI in 86% of cases.

### Use of carmustine wafers

Although 285 patients were felt suitable for maximal resection (i.e. > 90%), only 94 patients (33%) received carmustine wafers. In this group of patients, the median age was 61 (interquartile range 54 – 64), 62% were male, 77 patients (82%) had a KPS≥80 and 74 (79%) had a WHO score of 0 or 1. The median number of wafers used was 8 (range 3 – 8). For the group treated with carmustine wafers, 95% were glioblastomas or gliosarcomas compared to 88% of those not receiving carmustine. Only 3 of the 27 patients with an eligible WHO III tumour had carmustine wafers inserted.

Of the 191 patients suitable for maximal resection who did not receive carmustine wafers, 74 (39%) had intra-operative reasons why carmustine was not used, including 32 patients (17%) with large exposure of the CSF-ventricles caused by tumour resection, 30 patients (16%) where an intra-operative decision that maximal resection was not possible, 14 patients (7%) where there was no intra-operative cytological diagnosis to confirm high grade glioma and 3 cases (2%) where carmustine was not available from pharmacy. The other reasons for not using carmustine include the surgeon’s preference in 77 cases (40%), no MDT decision in 34 patients (18%) or funding issues in 7 cases (3%). A number of patients had multiple reasons for non-insertion of carmustine wafers. Six of the neurosurgical centres (35%) did not use carmustine wafers at all over the period of this study.

### Assessment of resection

The extent of resection was objectively assessed intra-operatively using 5-aminolevulinc acid fl uorescence surgery in 47 patients (16%), with 3D ultrasound guidance being used in another 3 (1%). Intra-operative MRI was not available at any of the centres involved in this study. Post-operative imaging (CT or MRI) was used to confirm maximal resection (i.e. >90%) in 63% of all patients, and 75% of patients receiving carmustine.

### Post-operative status at 48 hours

There was no change in either the KPS (77% with KPS≥80 vs. 78% pre-operatively) or WHO status (WHO ≤ 1 in 82% vs. 82% pre-operatively) at 48 hours. Of the 296 patients, 90% had no complications, and of the 29 with complications, 3 had more than one. Seven had a hemiparesis and/or aphasia, four had intracerebral haematomas, three had CSF leakage from the wound, two cerebral infarcts, two suspected wound infections, one seizure, one steroid induced mania and the remainder various systemic problems (e.g. pulmonary problems, difficult diabetic control and pyrexia). There was no difference in the incidence of early post-operative complications in those receiving and not receiving carmustine (*p* 0.84) (Table I). Nine complications were reported in the carmustine group. In eight cases, they were felt not related to the use of carmustine; in the ninth case, causality was not recorded.

**Table I:** Complications at 48 hours post-operatively

	All patients (*n* 296)	Carmustine wafers (*n* 94)	Non-carmustine group (*n* 202)
Any complication	29 (10%)	9 (10%)	20 (10%)
Brain swelling	5 (2%)	3 (3%)	2 (1%)
Pseudoabscess	0 (0%)	0 (0%)	0 (0%)
Wound leak	3 (1%)	1 (1%)	1 (0.5%)
Other complication	26 (9%)	8 (9%)	18 (9%)
1 complication	3 (1%)	2 (2%)	1 (0.5%)

The overall median length of stay was 4 days (interquartile range 3 – 7). There was no significant difference between those treated with carmustine wafers (median 4, interquartile range 2 – 6) and those not treated with carmustine wafers (median 5, interquartile range 3 – 8). Overall 28 patients (9%) were readmitted for a median of 11 days (range 5 – 21). There were no statistically significant differences between readmission for the group treated with carmustine (13% readmitted, 95% CI 6.0 – 19.5%; median duration 16 days, range 6 – 20.5) and those that did not receive carmustine (8% readmitted, 95% CI 4.2 – 11.6%; median duration 10.5 days, range 4.5 – 23.5).

### Adjuvant therapy

Follow-up data was recorded in 288 of all patients (97%) and 90 of the carmustine patients (96%). In total, 273 of all patients (95%) and 86 of the carmustine patients (96%) received radiotherapy following surgery. The median dose received was 60 Gy (range 15 – 60) given in a median of 30 fractions (range 2 – 31). The median interval between surgery and starting radiotherapy was 40 days (range 3 – 164 days). There was no difference between carmustine patients (median delay 41 days; range 14 – 152 days) and non-carmustine patients (median delay 40 days; range 3 – 164 days).

A total of 181 patients (63%) were prescribed concurrent temozolomide with their radiotherapy (Stupp protocol).^9^ Of these, 59 (63% of those receiving carmustine) had also been treated with carmustine. A number of 152 patients (84%) were prescribed adjuvant temozolomide and 50 had received carmustine wafers (i.e. 53% of the patients treated with carmustine wafers).

### Clinical status at 6 weeks following radiotherapy

By 6 weeks following radiotherapy, 67% of patients had a KPS≥80 (compared to 77% at 48 hours and 78% pre-operatively), and 76% had a WHO≤1 (compared to 82% pre-operatively and at 48 hours).

Review of surgical complications, summarised in Table II, at this time revealed that 262 patients had no surgical complication. Twenty-six patients (9%) had complications and seven of these had more than one. These included brain swelling (9), discharge from the wound (5), wound/brain infection (7), DVT/PE (5), focal deficits (2), seizures (2) and three deaths. Those patients having carmustine wafers did not have an increased risk of surgical complications compared to those patients not given carmustine (*p* 0.47, Fisher exact test). There was no increased complication risk in those patients treated with carmustine wafers and temozolomide compared to all other patients (*p* 1.0, Fisher exact test).

**Table II:** Complications at 6 weeks post radiotherapy

	All patients (*n* 288)	Carmustine wafers (*n* 90)	Non-carmustine group (*n* 198)
Any complication	26 (9%)	13 (14%)	13 (7%)
Brain swelling	9 (3%)	7 (8%)	2 (1%)
Pseudoabscess	0 (0%)	0 (0%)	0 (0%)
Wound leak	5 (2%)	3 (3%)	2 (1%)
Other complication	21 (7%)	9 (10%)	12 (6%)
> 1 complication	7 (2%)	5 (6%)	2 (1%)

Comparing the group receiving chemoradiotherapy and carmustine wafers with those treated with chemoradio-therapy alone, there was no statistically significant increase (*p* 0.16) in complications at 6 weeks post radiotherapy (4/122 for temozolomide alone versus 5/59 for temozolo-mide and carmustine wafers). Both groups had one wound leakage each.

## Discussion

In this study, we have shown that although 285 of the cohort fitted the NICE criteria as being suitable for insertion of carmustine wafers (patient ≥ 18 years, histologically confirmed high grade glioma and pre-operative assessment that >90% resection was possible), only 94 patients (33%) received it. For the 191 patients not receiving carmustine wafers, there were good intra-operative reasons in 79 of patients (41%) why they should not be used. In the remaining 58% of cases, the reason for not using carmustine was either due to surgeon’s preference or the lack of an MDT decision. No patient received carmustine wafers outside of the NICE criteria. This suggests compliance with NICE Guidance TA121 was good in relation to the management of patients receiving Gliadel, that is, selection of patients, but compliance across the United Kingdom was variable in terms of allowing patients access to the choice of treatments afforded by the NICE Guidance. The aim of the MDT process was to ensure best practice in the management of cancers. As a result, the implementation of NICE guidance was one of the MDT’s main roles. It is clear from our data that this has not been successful for the TA121 Technology Appraisal. Only one third of the units contributing to this study treated any patients with carmustine wafers. The underlying reasons for this variation remain unclear. Although we have information about a pre-operative decision to treat with or without carmustine wafers, we cannot be sure it was derived from a discussion in an MDT and, in particular, what influenced decision making. Since the publication of the Stupp Trial,^9^ this therapy has been regarded as the ‘new standard in care’.^10^ The role of carmustine wafers in primary treatment of malignant gliomas has subsequently been neglected because of uncertainties about the potential toxicity of a carmustine wafer + temozolomide-radiotherapy schedule as well as doubts about the statistical validity of the Phase III randomized controlled trial of Gliadel for primary malignant glioma.^11^ The latter doubt has been addressed by a recent Cochrane review that shows carmustine wafers do increase survival.^12^

There has been some confusion with NICE combining the appraisal of temozolomide and carmustine wafers. Some have concluded that the appraisal says these patients can be treated with temozolomide OR carmustine wafers. This is not the case. Both products were approved by NICE, and the appraisal states that ‘guidance does not relate to the sequential use of these treatments’. No Phase III trials exist but a range of recent studies that describe use of carmustine wafers followed by temozolomide-radiotherapy have addressed the problem of toxicity.10,^13–15^ The problem of combined therapeutic toxicity has also been addressed in an eclectic way. In some of these studies toxicity was greater but in others the combined treatment made no difference. There was also variability in median survival times between studies. Patient cohorts ranged from 28 to 44 patients. A Phase II safety study (GALA-5) Trial, ISRCTN 77105850) is underway to address this question. In our study, the use of carmustine wafers did not affect access to adjuvant oncological therapies and a particularly encouraging finding was that combining carmustine wafers with concomitant temozolomide and radiotherapy did not increase morbidity compared to resection and chemoradiotherapy alone.

Our data shows that there is no increase in complications at either 48 hours postsurgery or 6 weeks post-radiotherapy with carmustine wafers. We found similar postsurgical complication rates to those published by Westphal^6^ with 3% of patients developing wound leakage (compared to 5%) and 7% developing brain swelling (compared to 9.1% – although in the Westphal study, this was a late event occurring more than 6 months in 9 of 11 cases). There was also no increase in length of stay or readmission rate, and no increase in complications at 6 weeks post chemoradiotherapy if carmustine wafers were inserted.

Our study does highlight the lack of adjuvant technologies to assist resection of these tumours. Although imageguidance is commonplace in the United Kingdom, intra-operative ultrasound was used in only a small number of cases. 5-ALA, a technique shown in a multicentre Phase III trial to improve the extent of resection and thus prolong the time to progression^16^ was available in only three centres at the time of this study and was only used in a small number of patients. There was also a lack of post-operative imaging performed, either due to service restrictions or perceived lack of necessity even though it has been shown to be the only method to objectively assess the extent of resection.^17^

Although this study provides a snapshot of surgical neurooncology practice in the United Kingdom, the findings have to be tempered by the fact that over 60% of patients were recruited from four units. Even if we appreciate that only a quarter of glioblastoma patients might be eligible for carmustine wafers and would fit within the inclusion criteria of this study,^18^ the small number of patients recruited from some centres would suggest there has been selection bias in these centres. If we were to assume an incidence of 4 per 100 000 for high grade gliomas,^19^ we would expect 1280 patients diagnosed per year in the study population. As recruitment was probably only over a 2-year period and that only a quarter of patients might be eligible for carmustine wafers, the observed 296 patients is only 46% of what we would expect. This highlights the difficulty of recording such data in a clinical setting without the assistance of research nurses. In the four largest contributing centres, however, the observed 188 patients for this population is close to the expected value of 214 patients (88% of expected).

## Conclusions

This study has shown that there is great variability in the implementation of the NICE TA121 Technology Appraisal and that only a third of patients that would be suitable actually received carmustine wafers. Of those not receiving carmustine wafers, 60% were due to surgeon’s preference and lack of MDT decision. Barriers to implementation need to be identified.

## Participating centres and investigators in the UK-HGG Study Group

*Cambridge University Hospitals NHS Foundation Trust* (79), Stephen Price, *Lothian University Hospitals NHS Trust (Edinburgh)* (42), Ian Whittle, Shanne McNamara; *King ’ s College Hospital NHS Foundation Trust* (35), Keyoumars Ashkan; *Southampton University Hospitals NHS Trust* (32), Paul Grundy; *Royal Free Hampstead NHS Trust* (18) Lewis Thorne, Jane Baranowski; *The Newcastle upon Tyne Hospitals NHS Foundation Trust* (18) Joanne Lewis, John Crossman, Lynn Park; *University Hospital of North Staffordshire NHS Trust (Stoke on Trent)* (17) Jagmohan Singh, Catharine James; *Barking, Havering & Redbridge University Hospitals NHS Trust (Romford)* (11) Jonathan Pollock, Kim Grove; *Lancashire Teaching Hospitals NHS Foundation Trust (Preston)* (11) Charles Davies, Julie Law; *Brighton & Sussex University Hospitals NHS Trust (Haywards Heath)* (9) Giles Critchley, Ja-Eun Kim; *University Hospitals Birmingham NHS Foundation Trust* (8) Garth Cruickshank, Claire Goddard, Fred Berki; *Hull and East Yorkshire Hospitals NHS Trust (Hull)*(4) Shailandra Ashawal, Louise Baker Lynne Gill; *University Hospitals Coventry & Warwickshire NHS Trust (Coventry)* (3) Munchi Choksey, Ian Edwards; *NHS Tayside (Ninewells, Dundee)* (3) Sam Eljamel; *Abertawe Bro Morgannwg University Health Board (Swansea)* (2) Rupert Kett-White, Ann Hubschmid; *Imperial College Healthcare NHS Trust (Charing Cross)* (2) Kevin O’ Neill; *NHS Grampian (Aberdeen)*(2) Emmanuel Labram.
